# Chlorogenic acid exerts neuroprotective effect against hypoxia-ischemia brain injury in neonatal rats by activating Sirt1 to regulate the Nrf2-NF-κB signaling pathway

**DOI:** 10.1186/s12964-022-00860-0

**Published:** 2022-06-10

**Authors:** Yihui Zheng, Luyao Li, Binwen Chen, Yu Fang, Wei Lin, Tianlei Zhang, Xiaoli Feng, Xiaoyue Tao, Yiqing Wu, Xiaoqin Fu, Zhenlang Lin

**Affiliations:** 1 Department of Neonatology, The Second Affiliated Hospital and Yuying Children’s Hospital of Wenzhou Medical University, Wenzhou, China; 2 School of Second Clinical Medical, Wenzhou Medical University, Wenzhou, China

**Keywords:** Chlorogenic acid, Hie, Sirt1, Nrf2-NF-κB

## Abstract

**Background:**

Neonatal hypoxic-ischemic brain injury (HIE) is caused by perinatal asphyxia, which is associated with various confounding factors. Although studies on the pathogenesis and treatment of HIE have matured, sub-hypothermia is the only clinical treatment available for HIE. Previous evidence indicates that chlorogenic acid (CGA) exerts a potential neuroprotective effect on brain injury. However, the role of CGA on neonatal HI brain damage and the exact mechanism remains elusive.

Here, we investigate the effects of CGA on HI models in vivo and in vitro and explore the underlying mechanism.

**Methods:**

In the in vivo experiment, we ligated the left common carotid artery of 7-day-old rats and placed the rats in a hypoxic box for 2 h**.** We did not ligate the common carotid artery of the pups in the sham group since they did not have hypoxia. Brain atrophy and infarct size were evaluated by Nissl staining, HE staining and 2,3,5-triphenyltetrazolium chloride monohydrate (TTC) staining. Morris Water Maze test (MWM) was used to evaluate neurobehavioral disorders. Western-blotting and immunofluorescence were used to detect the cell signaling pathway. Malondialdehyde (MDA) content test, catalase (CAT) activity detection and Elisa Assay was used to detect levels of inflammation and oxidative stress. in vitro experiments were performed on isolated primary neurons.

**Result:**

In our study, pretreatment with CGA significantly decreased the infarct volume of neonatal rats after HI, alleviated brain edema, and improved tissue structure in vivo. Moreover, we used the Morris water maze to verify CGA’s effects on enhancing the learning and cognitive ability and helping to maintain the long-term spatial memory after HI injury. However, Sirt1 inhibitor EX-527 partially reversed these therapeutic effects. CGA pretreatment inhibited neuronal apoptosis induced by HI by reducing inflammation and oxidative stress. The findings suggest that CGA potentially activates Sirt1 to regulate the Nrf2-NF-κB signaling pathway by forming complexes thereby protecting primary neurons from oxygen-glucose deprivation (OGD) damage. Also, CGA treatment significantly suppresses HI-induced proliferation of glial.

**Conclusion:**

Collectively, this study uncovered the underlying mechanism of CGA on neonatal HI brain damage. CGA holds promise as an effective neuroprotective agent to promote neonatal brain recovery from HI-induced injury.

**Graphical Abstract:**

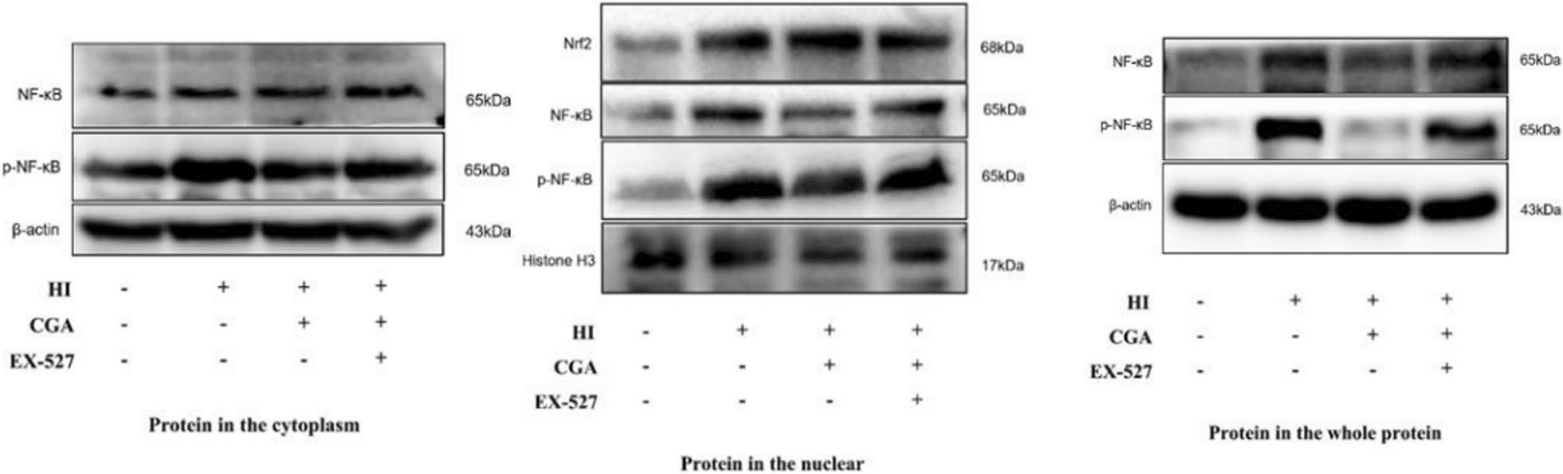

**Video Abstract**

**Supplementary Information:**

The online version contains supplementary material available at 10.1186/s12964-022-00860-0.

## Background

Neonatal hypoxic-ischemic encephalopathy is a brain injury that occurs in the perinatal period. Mounting evidence indicates that about 3 out of every 1,000 live births are likely to develop neonatal hypoxic-ischemic encephalopathy [1]. An ongoing concern is a high incidence and high mortality of HIE that needs to be solved urgently during the perinatal period [2]. Although scholars have deeply explored HIE, hypothermia treatment remains the only clinically recognized treatment method. Furthermore, the treatment window for mild hypothermia is short, and both the therapeutic effect and the nerve repair capacity for patients with severe neonatal ischemic hypoxic encephalopathy are limited [3]. Therefore, it would be imperative to uncover a safe and effective treatment method to assist in the diagnosis and treatment of brain injury with mild hypothermia therapy.

At low concentrations of antioxidants, an immature brain becomes highly susceptible to reactive oxygen and reactive nitrogen-mediated damage, and this is why the developing brain is extremely hypoxia sensitive. Studies have shown that neonatal hypoxic-ischemic encephalopathy involves different mechanisms, including oxidative stress [4], inflammation [5], apoptosis [6], autophagy [7], and so on. In particular, inflammation is implicated in the pathogenesis and pathophysiological changes of ischemic brain injury. Nuclear factor erythroid 2 related factor 2 (Nrf2), a key anti-inflammatory gene [8], directly regulates the expression of the HMOX1 gene encoded by the heme oxygenase HO-1 enzyme [9]. Evidence from in vitro and in vivo investigations has validated the role of the Nrf2-HO-1 signaling pathway in the inflammatory response. For instance, after Nrf2 knockdown, a study reported significantly increased expression of inflammation-related proteins COX-2, iNOS, IL-6, and TNF-α in the brains of Alzheimer's mice [10]. A similar investigation found elevated expression of HO-1 and NQO1 increased, providing evidence on the role of the Nrf2/HO-1 axis in inflammation [11]. Nuclear factor kappa-light-chain-enhancer of activated B (NF-κB) interacts with Nrf2 during oxidative stress. As such, increased NF-κB nuclear displacement will promote the expression of pro-inflammatory factors IL-1, IL-6, and TNF-α. There are different mechanisms through which Nrf2 can negatively regulate the nuclear displacement of NF-κB [12]. For instance, Nrf2 blocks the activation of NF-κB by reducing the level of intracellular ROS, and its up-regulation induces an increase in cellular HO-1 level, decreasing the expression of related enzymes to prevent IκB-α proteasome degradation and inhibit the nuclear translocation of NF-κB [13–15]. Moreover, to prevent the binding of CBP to Nrf2, NF-κB reduce the amount of free state that is the active state of cAMP-response-element-binding protein-binding protein (CBP), the transcriptional coactivator of Nrf2 [16]. Sirtuins are a family of NAD-dependent protein deacetylases, that contribute to the regulation of various cellular mechanisms, including redox, inflammation, and autophagy [17]. Recent experimental evidence indicates that SIRT1 play a part in the regulation of nuclear translocation of NF-κB, it activates the activation of Nrf2 and regulates the activity of NF-κB in HIE rats [18–20]. SIRT1 also regulates Nrf2 and inhibits the TLR4 pathway, thereby alleviating colitis-induced inflammation and pyrolysis [21, 22].

Chlorogenic acid (CGA) is mainly extracted from coffee but also Chinese medicine honeysuckle, chrysanthemum, honeysuckle, and other plants [23]. It has anti-inflammatory and antioxidant effects, therefore, exert a protective effect in cardiovascular-related diseases [24]. CGA has also been shown to promote metabolism [25] and improve aging [26], which is why it is widely used in food and medicine. Evidence from studies indicates that CGA regulates the Nrf2 pathway related to oxidative stress in cerebral ischemia-reperfusion (CI/R) injury, exerting a neuroprotective effect [27]. In neonatal rats with alcoholic brain injury, CGA was revealed to exert an anti-apoptotic effect by regulating the enzyme activity of caspase-3 [28]. Elsewhere, CGA pretreatment increased copper and zinc superoxide dismutase (SOD1) in the hippocampus following transient forebrain ischemia and inhibited the expression of pro-inflammatory factors, interleukin-4 (IL-4) and interleukin-13 (IL-13) [29].

Although there is evidence that CGA plays a role in neuroprotection, studies on its effect in neonatal hypoxic-ischemic encephalopathy are immature, and the specific mechanism remains to be determined. Therefore, this study explored the role of CGA in HI-induced neonatal hypoxic-ischemic encephalopathy and elucidated whether the SIRT1/Nrf2/HO-1 pathway mediates this process.

## Materials and methods

### Reagents

CGA (purity ≥98%) was purchased from Solarbio (Wuhan, China). Primary antibodies were purchased as follows: TNF-a (ab66579; Abcam, Cambridge, United Kingdom), iNOS (ab178945; Abcam, Cambridge, United Kingdom), β-Actin (#3700; Cell Signaling Technology, MA, United States), Histone H3 (#4499; Cell Signaling Technology, MA, United States), IkBa (#9242; Cell Signaling Technology, MA, United States), NF-κB (#8242; Cell Signaling Technology, MA, United States), p-NF-κB (#AF2006, affinity, United States), Nrf2 (#12721; Cell Signaling Technology, MA, United States), HO-1 (#43966; Cell Signaling Technology, MA, United States), IL-1β (#12703; Cell Signaling Technology, MA, United States), Sirt1 (DF6033, affinity, United States), SOD2 (AF5198, affinity, United States), cleaved-caspase-3(AF7022, affinity, United States), caspase-8 p18 (WL00659, wanleibio, China) and caspase-8 (WL03426, wanleibio, China). The secondary antibodies of Goat Anti-Rabbit IgG and Alexa Fluor®594 labeled were purchased from Bioworld (OH, United States). Dimethylsulfoxide (DMSO) was purchased from Sigma Chemical Co. (St. Louis, MO, United States). Fetal bovine serum, B27, neurobasal medium, L-glutamine (0.5 mM), and Dulbecco’s modified Eagle medium were purchased from Gibco (Grand Island, NY, United States). Malondialdehyde (MDA) content test kit was purchased from Solarbio (Beijing, China). A catalase (CAT) activity detection kit was purchased from Solarbio (Beijing, China). Cell-Counting Kit-8 (CCK-8) was purchased from Dojindo (Kumano, Japan). The nuclear stain 4′,6-diamidino-2- phenylindole (DAPI) was purchased from Beyotime (Shanghai, China). A nuclear and cytoplasmic protein extraction kit was purchased from Beyotime (Shanghai, China). Bovine serum albumin (BSA) was procured from Beyotime Biotechnology (Shanghai, China).

### Neonatal Hypoxic-Ischemic brain injury model and drug administration

Sprague Dawley (SD) rats (200-250g) were purchased from the Shanghai Zoological Center of the Chinese Academy of Sciences. Animal breeding and experiments were conducted as per the requirements of the Animal Breeding and Use Committee of Wenzhou Medical University. Adult SD rats mate freely, and male pups were used in the experiment on the 7^th^ day after birth (P 7). A modified Rice-Fannuzzi model was used as described previously [30], and isoflurane was applied to completely anesthetize and maintain P 7 pups. Subsequently, the left common carotid artery of P 7 young mice was separated within 5 min, ligated, and cut. The pups recovered with the mother 2 h after the operation. The pups rested enough and were placed in a humidified mixed gas chamber composed of 92% N_2_ and 8% O_2_ and ventilated at a flow rate of 3 L/min for 2 h. The above hypoxia equipment was placed in a constant temperature water bath at 37.5°C. We did not ligate the common carotid artery of the pups in the sham operation group since they did not have hypoxia, but the common carotid artery of the pups of the other groups were ligated and the pups were treated separately. After the end of hypoxia, all the pups were caged to continue feeding, awaiting follow-up experiments. For the next few days, the rats were given drugs daily as follows: The CGA pretreatment group was intraperitoneally injected with different concentrations of CGA (150, 300, or 600 mg/kg) immediately after hypoxia, with an interval of 24 h between each administration, and the young mice were euthanized to establish the most effective concentration. EX-527 (0.5mg/kg) 5ul each (2 mm rostral, 1.5 mm lateral to bregma, and 2.5 mm below the skull surface) was intracerebroventricularly injected, 30 min before hypoxia [31]. The needle was held for another 10 min after the injection is completed, and then withdrawn at a speed of 1 mm/minute.

### 2,3,5-triphenyltetrazolium chloride (TTC) staining

2,3,5-triphenyl tetrazolium chloride (TTC) staining method was employed to measure the volume of cerebral infarction based on the previous experimental procedure for assessing the area of cerebral infarction [32]. Brain tissues were collected from P7 young mice, 24 h after HI injury, stored at −20 °C for 15 min, and cut into approximately 2mm thick coronal sections. Then, the coronal brain slices were placed in 1% TTC (Sigma, USA) solution and incubated in a 37 °C oven in the dark for 30 min. Subsequently, the 11% TTC solution was discarded and the tissues were fixed overnight with 4% paraformaldehyde. Image-Pro Plus software was applied to measure the volume of cerebral infarction.

### Pathological staining

Brain tissue specimens were collected 7 days after HI injury, whereby rats were anesthetized with isoflurane, and the heart was perfused with 25 ml of sterile saline and further perfused with an equal volume of 4% paraformaldehyde. The brain specimen was immersed in 4% paraformaldehyde, stored at 4 °C for 24 h, and embedded in paraffin. Sections were cut at the thickness of 5 μm from the paraffin block to visually show the hemispherical integrity of functional neurons between the cerebral cortex and the hippocampus coronal slices. The brain sections were then deparaffinized, hydrated, and stained with HE or Nissl solution (Sohrab, Beijing, China). Finally, an optical microscope was employed to assess and record the results of histological staining. ImageJ software was used to analyze the results.

### Nuclear protein and cytoplasmic protein extraction

The nuclear protein was extracted for western blot detection of nuclear protein changes [33]. Appropriate amounts of 1mM PMSF cytoplasmic protein extraction reagents A and B were mixed at a 20:1 ratio to prepare a tissue homogenate, which was put in a homogenizer for tissue homogenization. Subsequently, the homogenized liquid was transferred to a 1.5mlep centrifuge tube, left to stand on ice for 15 min, and then centrifuged (1500 g for 5 min at 4 °C). The supernatant (the cytoplasmic protein) was collected, and the pellet was stored at −80 °C waiting for further processing. The precipitate was added to the cytoplasmic protein extraction reagent A containing 1 mM PMSF in a ratio of 1:10, shaken vigorously for 5 s, and left to stand on ice for 15 min. Following the addition of cytoplasmic protein extraction reagent B at a ratio of 20:1, mixture was shaken vigorously for 5 s, left to stand on the ice for 1 minute, and centrifuged (12,000g at 4 °C for 5 min). The supernatant (cytoplasmic protein) was collected to save the pellet and merged into the previous cytoplasmic protein. After complete aspiration of the supernatant, 50μl of nuclear protein extraction reagent containing 1mM PMSF was added for precipitation. The mixture was vortexed for 30 sec, then put in the ice bath, and vortexed again for 15-30 s every 2 min for 30 min. The mixture was centrifuged at 12,000 g at 4°C for 10 min, and the supernatant was collected as the nuclear protein.

### Western blot

The extracted cerebral cortex tissue or primary neurons were lysed in RIPA lysis buffer containing 1 mM PMSF. A tweezer was used to tear up the cerebral cortex tissue. The tissue was homogenized using a tissue homogenizer, fully sonicated on ice for 10 min, and then centrifuged (4 °C at 12,000 rpm at a speed of 20 min) to obtain the supernatant. Measurement of protein concentration was taken using the BCA kit (Beyotime) and the protein was prepared. Sodium dodecyl sulfate (SDS)-polyacrylamide separation gel electrophoresis was used to separate the protein, and then the bands were transferred to PVDF membrane (Millipore). After blocking with a 5% skimmed milk solution or a 5% BSA solution diluted with TBST for 4 h, the membrane was incubated with primary antibodies overnight in a refrigerator at 4°C: iNOS (1:1000), Sirt1 (1:1000), TNF-a (1:1000), NF-κB (1:1000), p-NF-κB (1:1000), β-actin (1:5000), IkBα (1:1000), IL-1β (1:1000), Histone H3 (1:1000), Nrf2 (1:1000), cleaved-caspase-3 (1:1000) and HO-1 (1:1000). Subsequently, TBST was washed thrice for 5 min each time and incubated with the corresponding secondary antibody (1:10,000) for 90 min. Afte washing with TBST for 3 times, the blots were visualized using an ECL Plus chemiluminescence reagent kit (RPN3243; Amersham Bioscience, Bensenville, IL, USA) and quantified by the Imaging System (Bio-Rad).

### Elisa assay

We washed rat brain tissue with PBS, added PBS at a ratio of 1:9 and homogenized the samples on ice. We centrifuged the homogenate obtained above at 12,000 rpm for 10 min at 4°C, and took the supernatant (100 μl) for analysis. The enzyme-linked immunosorbent assay (ELISA) (E-EL-R0012c, Elabscience) was used to detect the content of IL-1β in the brain tissue of rats in each group. We set up blank wells, standard wells and sample wells on the ELISA plate. We filled the blank well with 100 μl of standard/sample diluent. The standard wells contained 100 μl of standard samples diluted in multiples, and the sample wells contained 100 μl of experimental samples. Then the plate was incubated at 37°C for 90 min. After being added the biotinylated antibody working solution (100 μl/well), the plate was incubated at 37°C for 60 min, followed by being washed with washing solution 3 times. Then the plate was incubated at for 30 min with 100 μl horseradish peroxidase conjugate solution. Next we washed it with washing solution for 5 times, then added 90μl of substrate solution to each well, and incubated it for 15 min at 37°C. Finally, we added 50μl of stop solution, and immediately measured the optical density of IL-1β at 450 nm wavelength.

### Malondialdehyde (MDA) content test

Lipid peroxidation level was detected based on the level of MDA [34]. Rats were euthanized after deep anesthesia with isoflurane three days after the HI injury, and the cerebral cortex tissues of the young rats were isolated and stored at -80°C for subsequent analysis. We weighed about 0.1g of tissue of each group, added 1mL of extracting solution to homogenize in ice bath, centrifuged it at 8000g at 4℃ for 10min, and took the supernatant for next testing. Subsequently, the MDA was extracted and placed in a microplate reader to detect the absorbance at wavelengths of 450nm, 532nm, and 600nm. Finally, according to the MDA content (nmol/g mass) =5× (12.9× (ΔA532 -ΔA600)-2.58×ΔA450) ÷ W. W: sample mass, g; ΔA450=Δ450 measurement-Δ450 blank; ΔA532=Δ532 measurement-Δ532 blank; ΔA600=Δ600 measurement-Δ600 blank, we measured the concentration of MDA.

### Catalase (CAT) activity detection

Catalase (CAT) is the most important hydrogen peroxide scavenging enzyme in the body [35, 36]. Using the catalase (CAT) activity detection kit as per the manufacturer’s instructions and a microplate reader, we detected the CAT content according to the change in absorbance over time at a wavelength of 240nm.

### Immunofluorescence staining

Coronal brain tissue slices were stained with GFAP. Briefly, the slices were dried in an oven at 65°C for 3 h and immersed in xylene to deparaffinize for 20 min. The slices were hydrated in gradient alcohol and washed thrice with PBS (5 min each time). Following antigen retrieval with sodium citrate, slices were washed thrice with PBS (5 min each time), diluted with 0.3% Triton X-100 (PBS), and reacted for 15 min at room temperature. We washed slices 3 times with PBS for 5 min each time, blocked them with 10% goat serum (PBS), and then incubated slices overnight at 4°C with GFAP (1:200) primary antibody. On the second day, after 3 times wash with PBS, slices were exposed to a secondary antibody (1:200) labeled with Alexa Fluor® 594for 1.5 h. After washing thrice with PBS (5 min each time), they were incubated with DAPI for 10 min, washed with PBS, and mounted with an anti-quenching agent. The samples were taken and preserved in an Olympus fluorescence microscope (Tokyo, Japan). ImageJ software was employed to determine the fluorescence intensity.

### Morris water maze test (MWM)

The Morris Water Maze Test (MWM) is a behavioral experiment for determining the cognitive function of animals [37]. We applied the MWM test, 21 days after the HI injury, to evaluate the learning and memory abilities of experimental animals, whereby they were put to swim to find a platform hidden underwater. A black circular pool with a diameter of 140 cm and a height of 50 cm was prepared in a room protected from noise and light. The water depth of the pool was 1 cm higher than the movable platform. Water was dyed black with non-toxic black ink and the pool was divided into four equal quadrants. The rats were trained for 6 days, after which the platform was removed. The swimming route, incubation time, and the number of times on stage were recorded. The experiment was performed by the SLY-WMS Morris water maze experiment system.

### Primary cortical neuron extraction and culture

Primary cortical neurons were extracted from the cerebral cortex of SD neonatal rats (P 0). Briefly, the newborn rat was immersed in 75% alcohol for 20 min, the brain was isolated to separate the cerebral cortex and washed thrice with PBS. Then we used 0.25% papain-EDTA solution to digest cerebral cortex tissue at 37°C for 15 min. After centrifugation of the final product above, the pellet was introduced to a 6-well cell culture plate coated with poly-D-lysine according to 1x105, incubated (5% CO_2_ at 37°C for 6 h). The culture medium was replaced with 2% B27, 0.5 Neural basal medium of mM L-glutamine, penicillin/streptomycin antibiotics.

### Cell viability

The cytotoxicity of CGA to primary cortical neurons was detected by the CCK-8 kit. Briefly, primary cortical neurons were extracted and inoculated in 96-well plates (8,000 cells/well). Then, the cells were treated with CGA in a concentration gradient of 0,100,200,300, and 400nM for 24 h. Finally, 10 μl of CCK-8 solution was added to each well of the 96-well plate and incubated at 37°C for 1.5 h. A microplate reader (Leica Microsystem, Germany) was used to detect the absorbance of each well at a wavelength of 450 nm.

### Immunofluorescence cell staining

We cultured primary cortical neurons for 7 days and then performed OGD on primary cortical neurons. After that, the cell slides were washed 3 times with PBS for 5 min each time. Then, after fixing with 4% cell fixative for 15 min, we washed them 3 times with PBS for 5 min each time. Then we diluted them with 0.3% Triton X-100 (PBS) for 15 min at room temperature. We washed slices 3 times with PBS for 5 min each time, blocked them with 10% goat serum (PBS), and then incubated slices overnight at 4°C with NF-κB (1:200) primary antibody. On the second day, after 3 times wash with PBS, slices were exposed to a secondary antibody (1:200) labeled with Alexa Fluor® 594for 1.5 h. After washing thrice with PBS (5 min each time), they were incubated with DAPI for 10 min, washed with PBS, and mounted with an anti-quenching agent. The samples were taken and preserved in an Olympus fluorescence microscope (Tokyo, Japan). ImageJ software was employed to determine the fluorescence intensity.

## Results

### Chlorogenic acid attenuates hypoxic-ischemic brain injury in neonatal rats

**Fig. 1:**
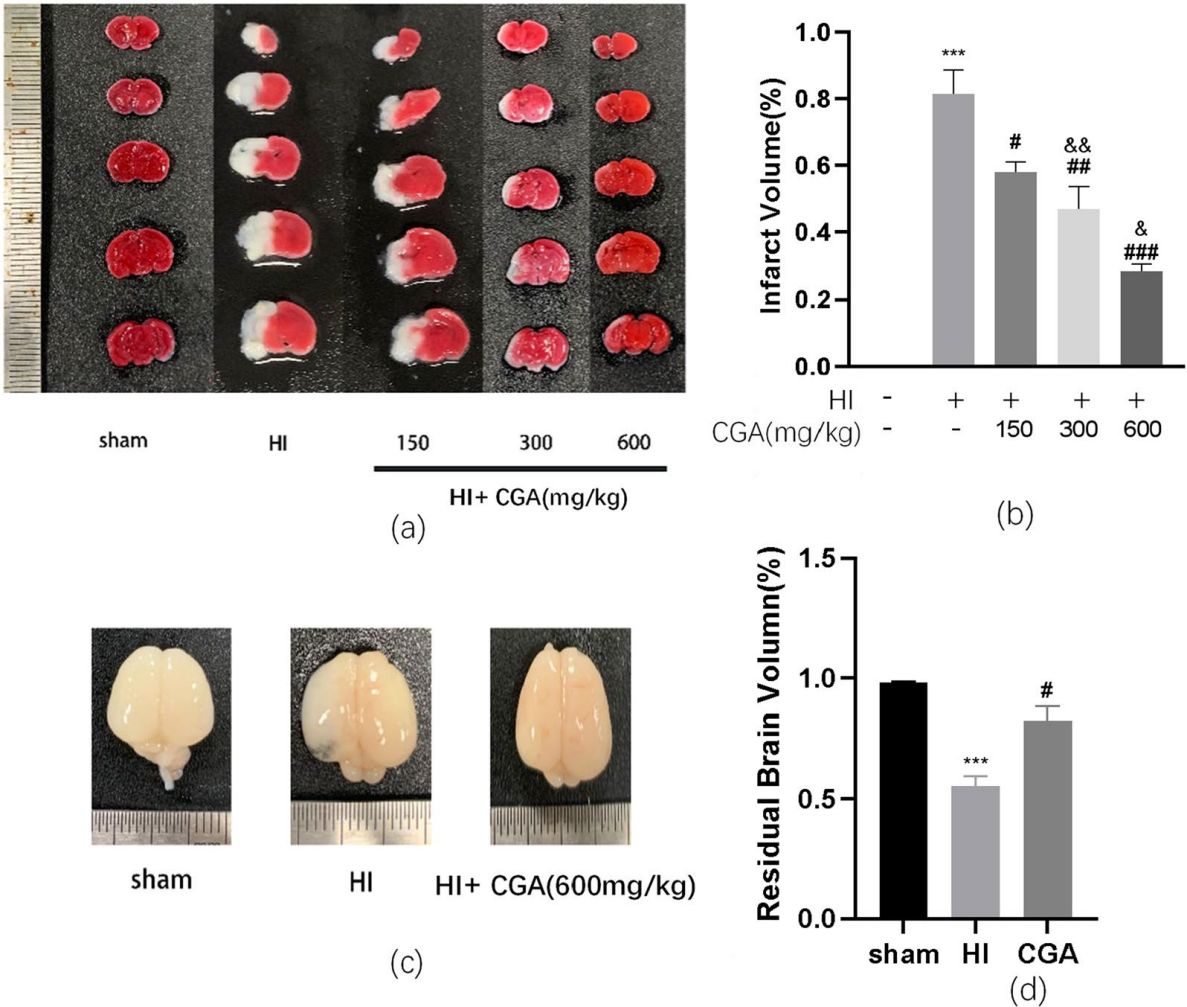
Chlorogenic acid-induced attenuation of hypoxic-ischemic brain injury in neonatal rats. **a** Representative images of TTC-stained coronal brain sections 24 h after HI brain injury with/without treatments. **b** Calculation of the infarct area depicted by TTC staining. ∗∗∗*P* < 0.001 vs. the sham group. #*P* < 0.05, ##*P* < 0.01 and ###*P* < 0.001 vs. the HI group. &*P* < 0.05, &&*P* < 0.01 vs. the CGA (150mg/kg) group. n = 4. **c** The brains isolated from each group 7days after HI brain injury. **d** The ratio of residual brain volume calculated in each group. ∗∗∗*P* < 0.001 vs. the sham group. #*P* < 0.05 vs. the HI group. n = 5

Rats were intraperitoneally injected with CGA at the range of concentrations (150, 300, 600 mg/kg), every 24 h for 3 consecutive days to investigate the role of CGA in the HI brain injury process and to establish the most effective animal administration dose. According to TTC staining results and the quantitative analysis of cerebral infarction volume, the 150, 300, 600 mg/kg doses of CGA effectively decreased the volume of cerebral infarction (Fig. 1a, b); notably, the therapeutic effect increased with an increase in the concentration. Therefore, we selected a concentration of 600 mg/kg for subsequent experiments. At the same time, the general brain changes 7 days after the HI injury, were assessed and compared among groups. The HI group was characterized by more edema and liquefaction as compared to the sham operation group (Fig. 1c, d). Furthermore, brain damage in the CGA (600mg/kg) pretreatment group was significantly less compared to the HI injury group (Fig. 1d). These data strongly demonstrate that CGA pretreatment can alleviate the brain damage of newborn rats after HI injury.

### Chlorogenic acid-induced protection of brain post-hypoxic-ischemic brain injury by decreasing inflammation and oxidative stress

**Fig. 2:**
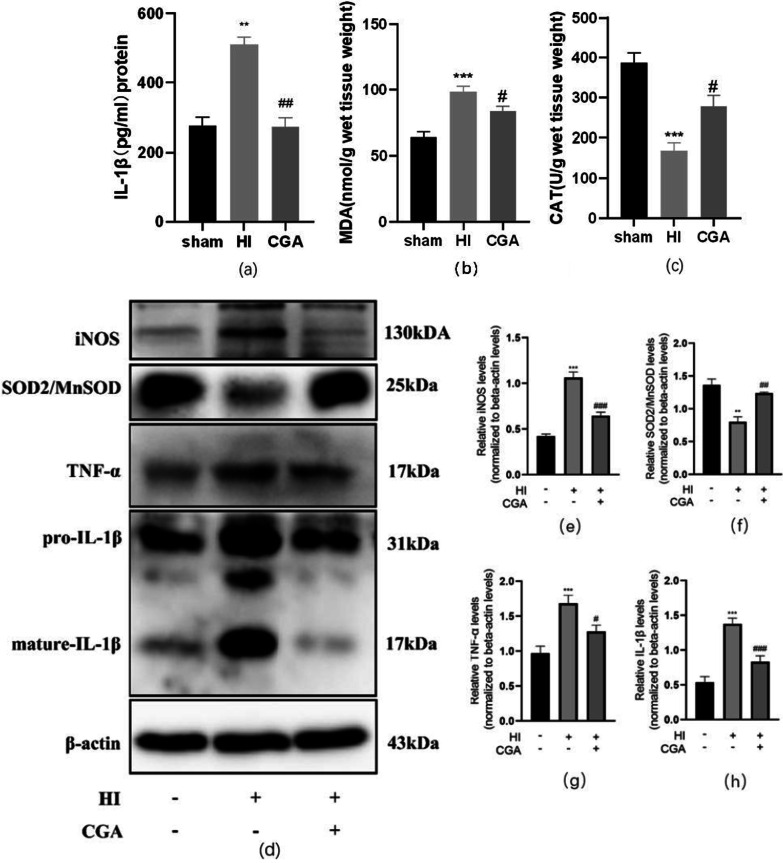
Chlorogenic acid-induced protection of brain post-hypoxic-ischemic brain injury via the down-regulation of expression of inflammatory and oxidative stress levels. **a** The IL-1 β level in brain tissue 24 h after HI brain injury measured by Elisa kit. ∗*P* < 0.05 vs. the sham group. ##*P* < 0.01 vs. the HI group. n=3. **b** The MDA level in brain tissue 24 h after HI brain injury by MDA kit. ∗∗∗*P* < 0.001 vs. the sham group. #*P* < 0.05 vs. the HI group. n=3. **c** The CAT level in brain tissue 24 h after HI brain injury by CAT kit. ∗∗∗*P* < 0.001 vs. the sham group. #*P* < 0.05 vs. the HI group. n=3. **d** Western blot detection of the protein levels of iNOS, SOD2/MnSOD, TNF-α, pre-IL-1β and mature- IL-1β 24h after HI injury. **e–h** Quantification of western blot data of iNOS, SOD2/MnSOD, TNF-α, and mature-IL-1 β. ∗∗*P* < 0.01 and ∗∗∗*P* < 0.001 vs. the sham group. #*P* < 0.05, ##*P* < 0.01 and ###*P* < 0.001 vs. the HI group. n = 3

Previous evidence indicates that CGA can promote the body's anti-inflammatory and anti-oxidative stress effects. Here, using ELISA, we detected pro-inflammatory factor, IL-1β expressed in brain tissue. Results demonstrated significantly increased IL-1β levels after HI injury. Notably, after CGA injection, we observed significantly reduced the expression level of IL-1β in brain tissue (Fig. 2a). Moreover, the level of lipid oxidation in the brain tissue was detected using the MDA kit. It was revealed that the level of MDA in the brain tissue of the sham operation group was 63.92±10.37nmol/g, while the average level of MDA in the brain tissue of the HI group was 98.48±10.01nmol/g. Moreover, the average level of MDA in the brain tissue of the CGA pretreatment group was 83.44±10.00nmol/g (Fig. 2b). CAT detection of the level of H_2_O_2_ elimination enzymes showed that the level of CAT (catalase) in the brain tissue of the sham operation group was 387.25±59.61 U/g, the level of CAT in the brain tissue of the HI group was 168.58±50.29 U/g, and the level of CAT in the brain tissue of the CGA pretreatment group was 278.16±67.07 U/g (Fig. 2c). The tissue proteins of the cerebral cortex of young rats were extracted 3 days after injury, followed by western blot detection of the expression levels of pro-inflammatory factors IL-1β, iNOS, TNF-α, and SOD2/MnSOD. Results demonstrated that, after HI injury, the levels of pro-inflammatory factors pre-IL-1β, mature- IL-1β, iNOS, and TNF-α increased significantly, while the levels of SOD2/MnSOD decreased significantly. Compared with the HI injury group, the CGA group exhibited significantly lower levels of pro-inflammatory factors pre-IL-1β, mature- IL-1β, iNOS, and TNF-α and significantly higher levels of SOD2/MnSOD. These data demonstrate the potential neuroprotective effects of CGA by reducing inflammation and oxidative stress (Fig. 2d, h).

### Chlorogenic acid exerts a neuroprotective effect in hypoxic-ischemic brain injury by activating Sirt1 to regulate the Nrf2-NF-κB signaling pathway

**Fig. 3:**
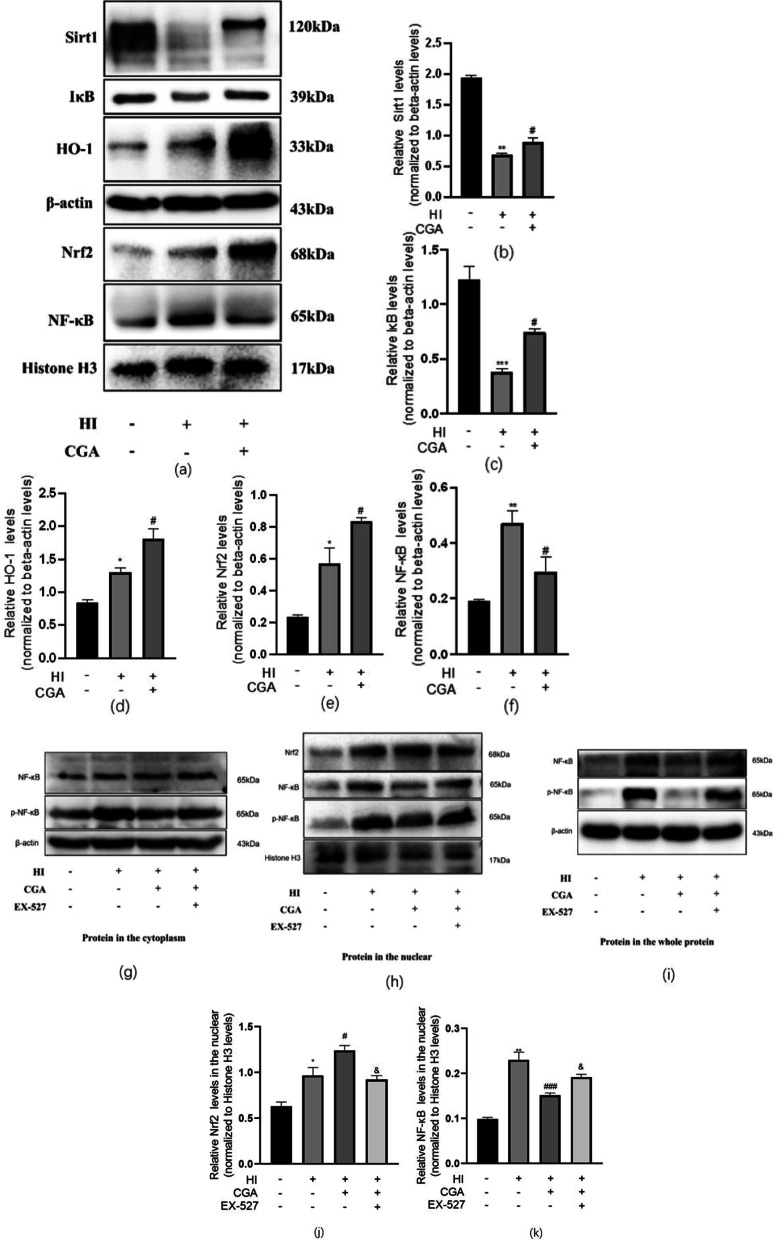
The neuroprotective role of CGA in hypoxic-sschemic brain injury through activation of Sirt1 to regulate the Nrf2-NF-κB signaling pathway. **a** Representative results from Western blot showing the protein levels of Sirt1, IκB, and HO-1 in cytoplasm, Nrf2 and NF-κB in nucleus 24h after HI injury. **b**–**f** Quantification of western blot data of Sirt1, IκB, HO-1, Nrf2, NF-κB. ∗*P* < 0.05, ∗∗*P* < 0.01 and ∗∗∗*P* < 0.001 vs. the sham group. #*P* < 0.05, ##*P* < 0.01 and ###*P* < 0.001 vs. the HI group. n = 3. **g** Representative results from Western blot showing the protein levels of NF-κB and p-NF-κB in the cytoplasm 24h after HI injury. **h** Representative results from Western blot showing the protein levels of NF-κB, p-NF-κB and Nrf2 in the nuclear 24h after HI injury. **i** Representative results from Western blot showing the protein levels of NF-κB and p-NF-κB in the whole protein 24h after HI injury. **j**–**k** Quantification of western blot data of Nrf2, NF-κB in the nuclear. ∗*P* < 0.05, ∗∗*P* < 0.01 vs. the sham group. #*P* < 0.05, ##*P* < 0.01 vs. the HI group. &*P* < 0.05, vs. the CGA pretreatment group. n = 3

Studies have revealed that CGA exerts anti-inflammatory effects by activating Nrf2-NF-κB during cerebral ischemia-reperfusion injury [38]. Sirt1 is a key regulator of the Nrf2-NF-κB signaling pathway. We explored the effect of CGA on Nrf2-NF-κB via Western blot analysis. Nuclear protein detection results indicated that HI damage could promote the nuclear transfer of Nrf2, and increase the nuclear levels level of NF-κB (Fig. 3a, e–f). In the CGA pretreatment group, the nuclear levels level of Nrf2 was higher than that of the HI group, while the level of NF-κB in the nucleus was significantly lower than the HI group. Cytoplasmic protein analysis demonstrated that, in the HI group, the levels of HO-1 increased while the levels level of Sirt1 and IκB decreased significantly. The levels of HO-1, Sirt1, and IκB were all higher in the CGA pretreatment group as compared to those of the HI injury group (Fig. 3a–d). After using the selective inhibitor of Sirt1, EX-527 in the pretreatment group, the level of nuclear internalization of Nrf2 was significantly lower than that of the CGA pretreatment group. And the levels of nuclear internalization of NF-κB and p-NF-κB were higer than that of the CGA pretreatment group. In the cytoplasm, the expression level of NF-κB did not change significantly, while the expression level of p-NF-κB increased significantly. In total protein of the tissue, the expression level of NF-κB increased significantly, and the expression level of p-NF-κB increased significantly. The selective inhibitor of Sirt1, EX-527, counteracted the neuroprotective effect of CGA through Nrf2-NF-κB (Fig. 3g–k).

### Chlorogenic acid attenuates apoptosis induced by HI injury

**Fig. 4:**
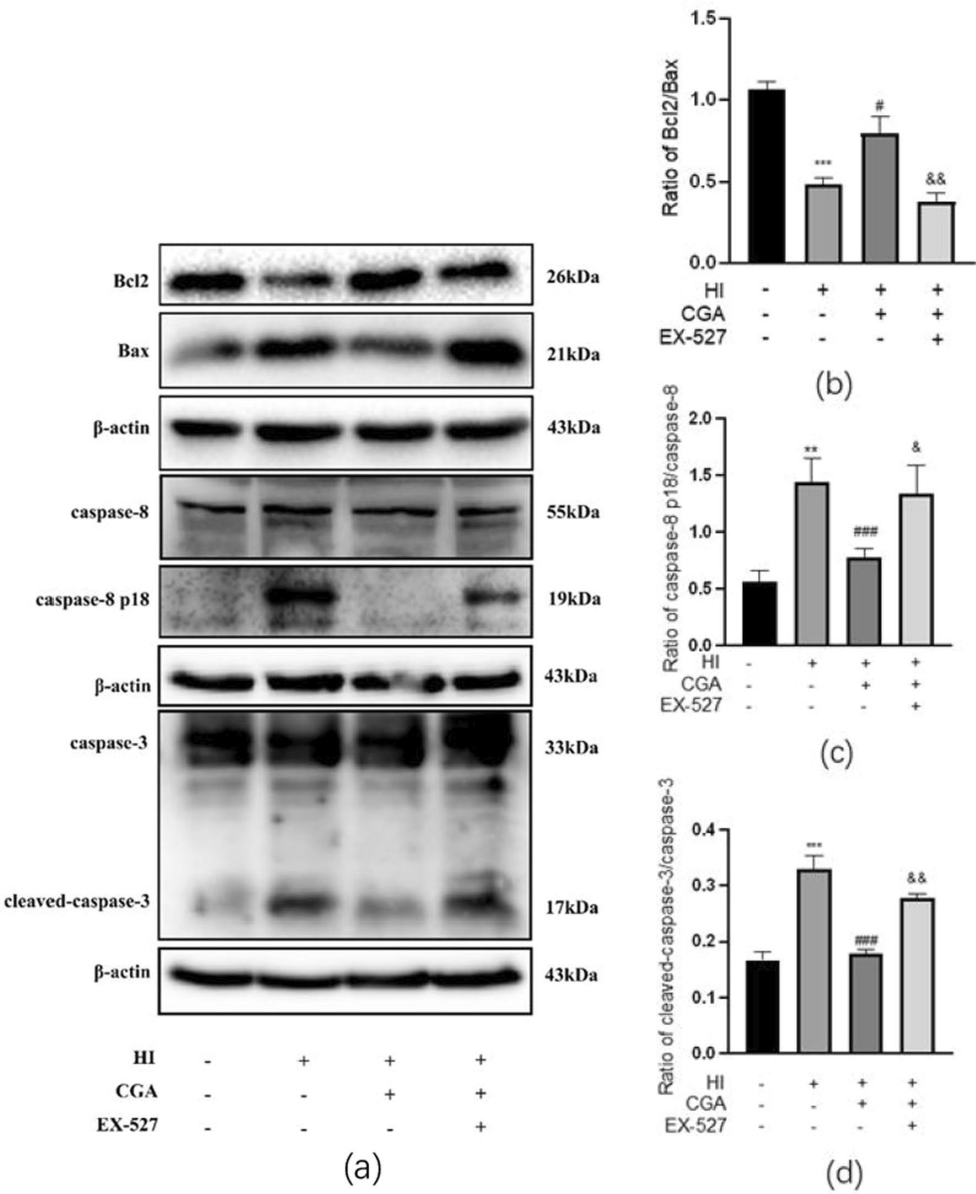
Chlorogenic acid (CA) treatment reduces HI-induced neuronal apoptosis. **a** Western blot evaluation of the protein levels of Bcl2, Bax, caspase-8, caspase-8 p18, cleaved-caspase-3 and caspase-3 24h after HI injury. **b** Quantification of western blot data of Bcl2/Bax. **c** Quantification of western blot data of caspase-8 p18/caspase-8. **d** Quantification of western blot data of cleaved-caspase-3/caspase-3. ∗∗*P* < 0.01, ∗∗∗*P* < 0.001 vs. the sham group. #*P* < 0.05, ###*P* < 0.001 vs. the HI group. &*P* < 0.05, &&*P* < 0.01 vs. the CA treatment group. n = 3

To detect the changes in the levels of apoptosis in each group, total protein was extracted from each group 24h after HI, followed by western blot detection of the ratio of Bcl2/Bax, ratio of caspase-8 p18/caspase-8 and ratio of cleaved-caspase-3/caspase-3 in each group. Results showed significant reduction of Bcl2/Bax in the HI group. The ratio of caspase-8 p18/caspase-8 and cleaved-caspase-3/caspase-3 significantly increased in the HI group. Significantly, the Bcl2/Bax ratio of the CGA treatment group was higher as compared to that of the HI group. The caspase-8 p18/caspase-8 ratio of and the cleaved-caspase-3/caspase-3 ratio of the CGA treatment group was lower as compared to that of the HI group. The selective inhibitor of Sirt1, EX-527, reversed the anti-apoptotic effect of CGA treatment (Fig. 4a–d).

### Chlorogenic acid improves hypoxic-ischemic brain injury-induced brain tissue structural damage

**Fig. 5:**
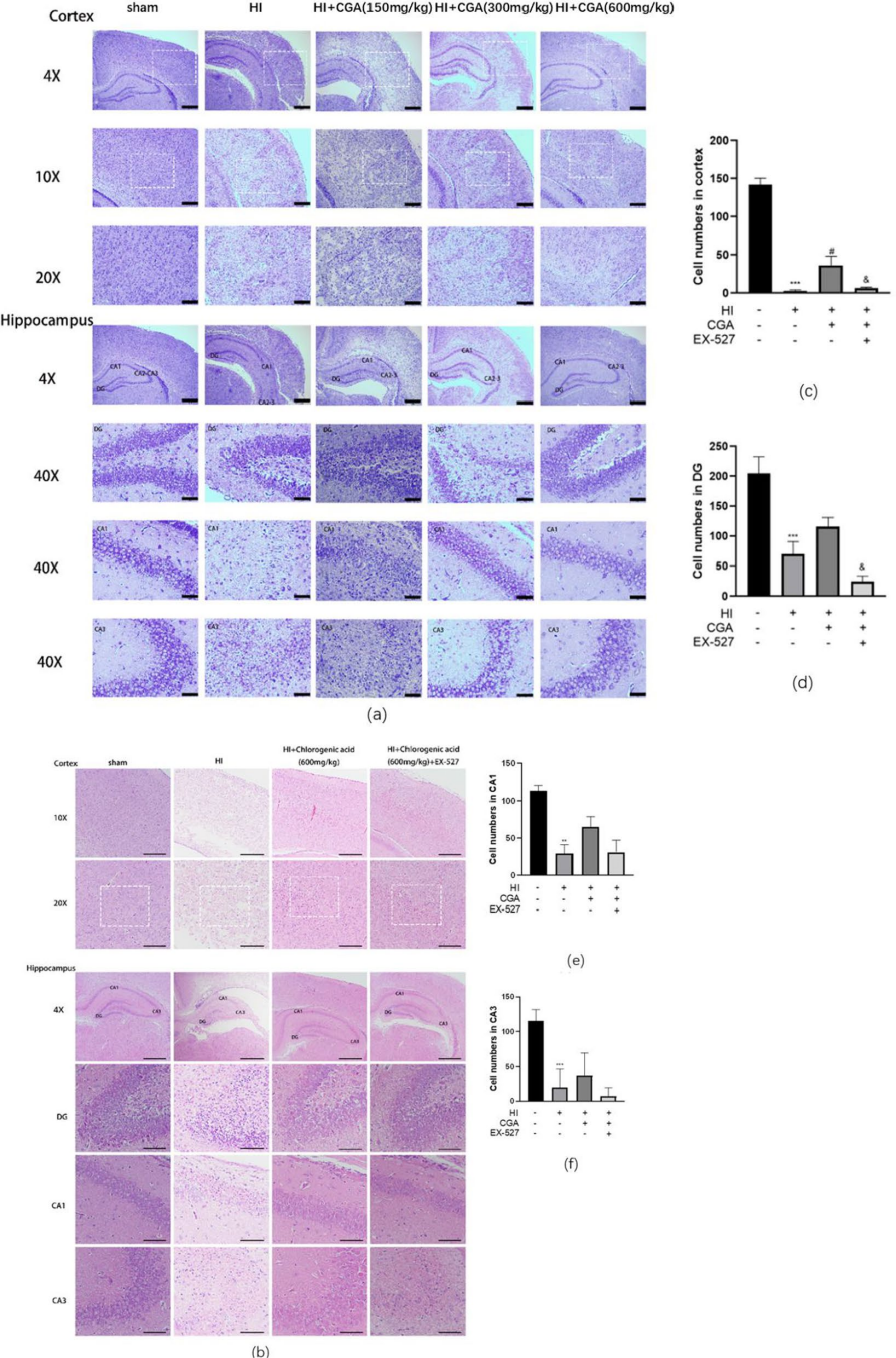
Chlorogenic acid improves hypoxic-ischemic brain injury-induced brain tissue structural damage. **a** Representative images of HE staining in the cortex, hippocampus CA1 region, hippocampus CA3 region, and DG region 7 days after HI injury. Scale bars represent 200μm in the images of 4X; Scale bars represent 100μm in the images of 10X; Scale bars represent 50μm in the images of 20X. Scale bars represent 20μm in the images of 40X. **b** Representative images of Nissl staining in the cortex, hippocampus CA1 region, hippocampus CA3 region, and DG region 7 days after HI injury. Scale bars represent 200μm in the images of 4X, Scale bars represent 100μm in the images of 10X, Scale bars represent 50μm in the images of 20X. Scale bars represent 20μm in the images of 40X. **c–f** Cell numbers in the cortex of the images of 10X, hippocampus CA1 region, hippocampus CA3 region, and DG region in each group,results of treatments are based on the 600mg/kg of CGA. ∗∗∗*P* < 0.001 vs. the sham group. #*P* < 0.05, ##*P* < 0.01 and ###*P* < 0.001 vs. the HI group. &*P* < 0.05, &&*P* < 0.01, &&&*P* < 0.001 vs. the CA treatment group. n = 3

The protective effect of CGA on neurons was assessed via HE staining and Nissl staining. By comparing the Nissl bodies of the HI injury group and the sham operation group, the cerebral cortex and hippocampus (DG area, CA1 area, CA3 area) of the sham operation group had large Nissl bodies mostly arranged close to the nucleus, while the HI group was almost invisible to Nissl body (Fig. 5a). Assessment of the stained sections of pathological tissues revealed an apparent left hemibrain injury in the HI injury group, especially in the cerebral cortex and hippocampus (DG area, CA1 area, CA3 area), the cell arrangement was disordered, and most neurons died. Compared the number of normal neurons in the selected area of the cerebral cortex in each group, we observed there were almost no normal neurons in the HI injury group. Moreover, the number in the images of hippocampal DG region, CA1 region, and CA3 region were counted and the number of cells in the DG, CA1 and CA3 areas decreased significantly after HI injury. CGA treatment increased the number of normal neurons and Nissl bodies with the increase in concentration, and a gradual recovery of the changes in cell structure was reported with the increase in dose. Of note, the selective inhibitor of Sirt1, EX-527, reversed the therapeutic effect of CGA (Fig. 5b–f).

### Chlorogenic acid treatment suppresses HI-induced activation of astrocyte in the neonate brain

**Fig. 6:**
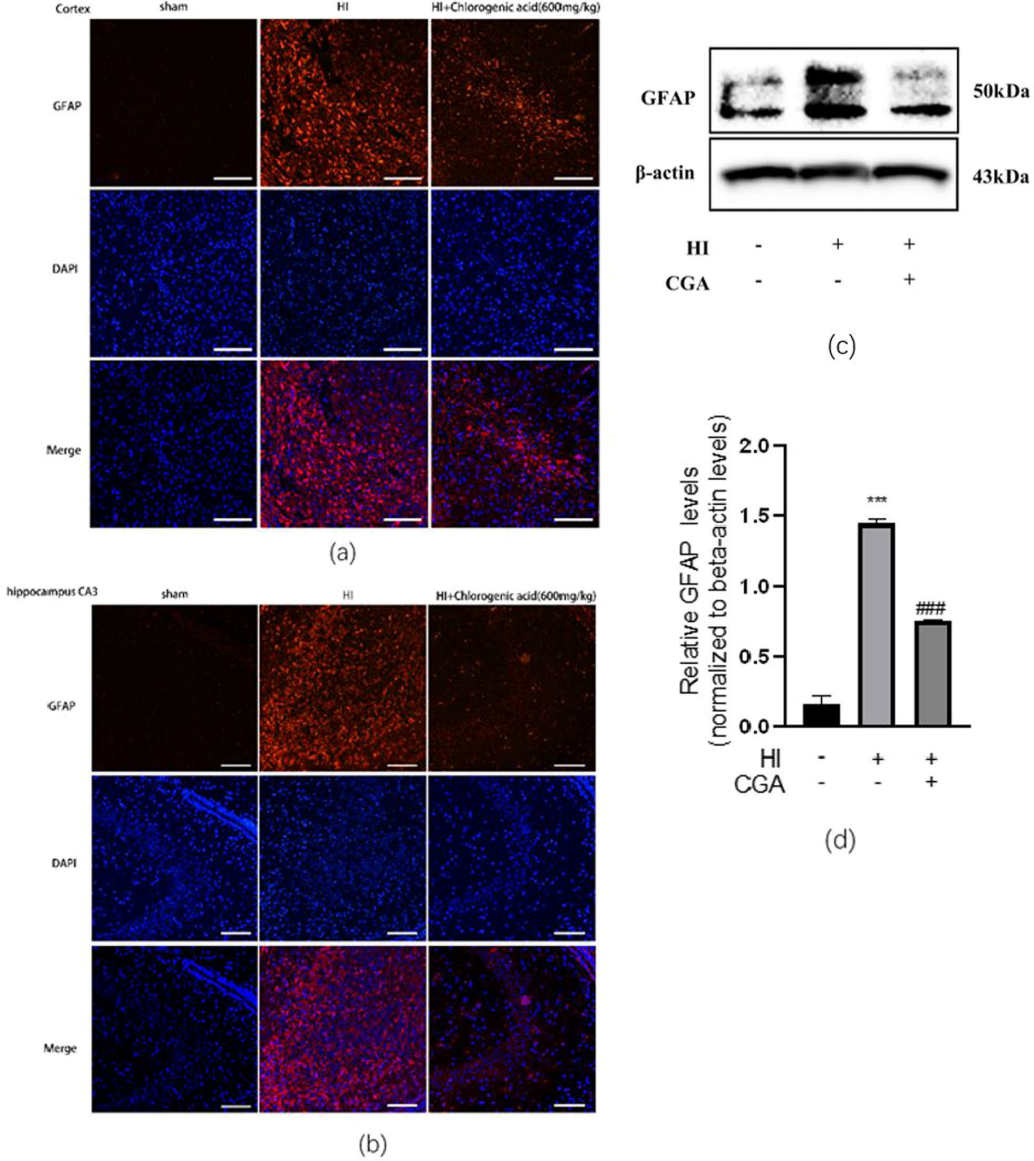
Chlorogenic acid treatment suppressed HI-induced activation of astrocyte in the neonate brain. (a, b) Representative immunofluorescent staining for GFAP expression in the cortex **a** and hippocampus CA3 region** b** from each group at 7days post-HI. Scale bar = 50 μm. n =3** c** Western blot evaluation of the protein levels of GFAP 1 day after HI.** d** Quantification of western blot data of GFAP. ∗∗∗*P* < 0.001 vs. the sham group. #*P* < 0.05 vs. the HI group. n = 3

GFAP is a crucial indicator of astrocyte activation. Here, GFAP expression in each group was assessed by tissue immunofluorescence staining. Experimental results demonstrated significantly high expression of GFAP in the cerebral cortex and hippocampal CA3 area after HI injury, and the astrocytes in these two areas were significantly enlarged and densely distributed, and the cell branches increased. In the HI+CGA group, GFAP expression was significantly reduced, the activation of astrocytes was blocked, and the branches of astrocytes decreased (Fig. 6a, b). At the same time, western blot detection of cerebral cortex tissue proteins,24 h after HI injury, revealed that GFAP levels after HI+CGA treatment was consistent with the results in immune tissue fluorescence (Fig. 6c, d).

### Chlorogenic acid ameliorates the long-term learning and cognitive function of rats with hypoxic-ischemic brain injury

**Fig. 7:**
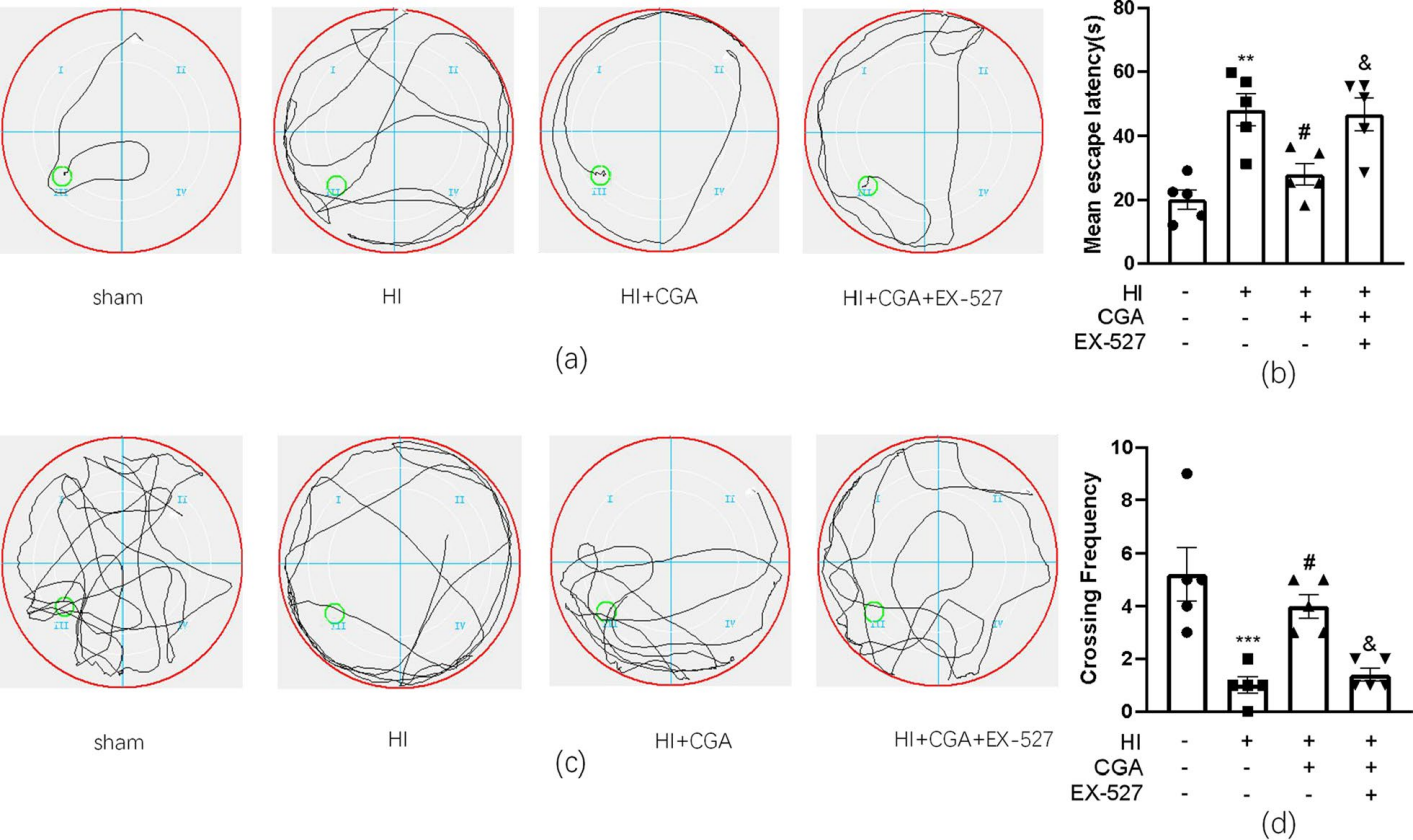
Chlorogenic acid ameliorates the long-term learning and cognitive function of rats with hypoxic-ischemic brain injury **a** Representative of swim route traces of rats from different groups. **b** Quantitative analysis of mean escape latency of Morris Water Maze tests in different groups of rats. **P < 0.01 vs. the sham group; #*P* < 0.05 vs. the HI group; &*P* < 0.05 vs. the HI + CA group. n = 5. **c** Representative images of swim route traces of rats from different groups after removal of the platform. **d** Quantitative analysis of the frequency of crossing the original platform location in the 60 s. ***P < 0.001 vs. the sham group; #*P* < 0.05 vs. the HI group; &*P* < 0.05 vs. the HI + CA group. n = 5

We evaluated the effect of CGA pretreatment on learning and cognitive function after HI injury by conducting the Morris water maze experiment. Data acquired in the spatial acquisition test revealed that after HI injury, the learning ability of rats decreased and the average escape latency of HI injury was longer as compared to that of the sham group. However, the average escape latency of rats in the HI + CGA group was significantly shortened, a trend that could be reversed by EX-527, the selective inhibitor of Sirt1 (Fig. 7a, b). In the reference memory test, the platform was removed after the last training on the 6th day. After 24 h, to investigate the spatial memory ability of the rats, we counted the number of times the rats crossed the platform after the platform was removed. In the CGA pretreatment group, we reported a significant increase in the number of bench crossings; however, EX-527 could reduce this effect (Fig. 7c, d).

### Chlorogenic acid decreases inflammation and oxidative stress in primary cortical neurons, induced by oxygen and glucose deprivation

**Fig. 8:**
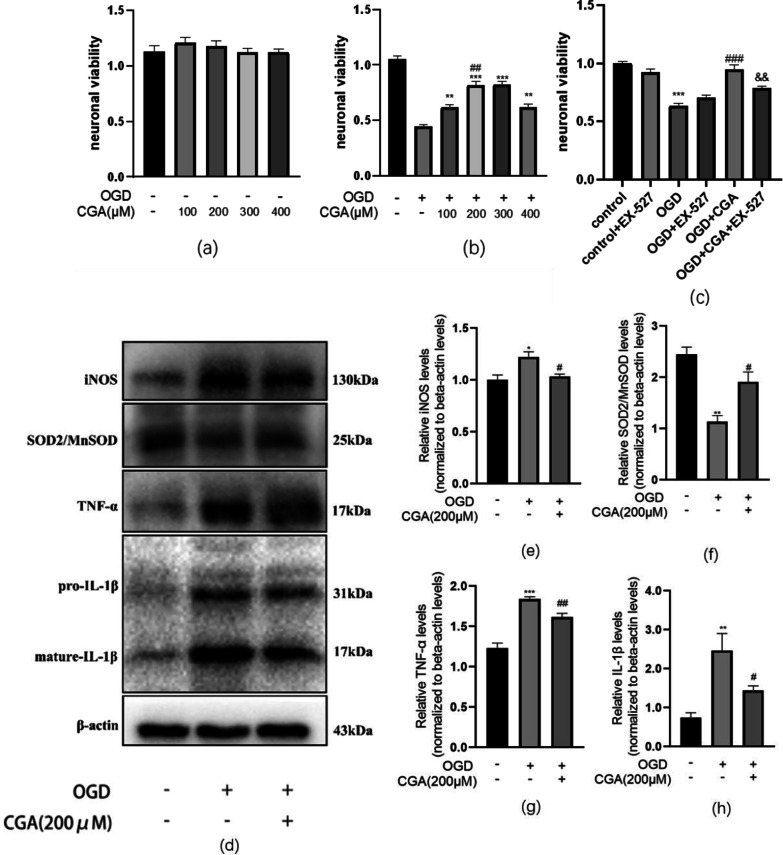
Chlorogenic acid decreases inflammation, oxidative stress of primary cortical neurons induced by oxygen and glucose deprivation. **a** Primary cortical neurons, treated with different concentrations of CGA without OGD; cell viability was assessed by CCK8. n = 3. **b** Primary cortical neurons pretreated with different concentrations of CGA before OGD; cell viability was assessed by CCK8. n = 3. **c** The effect of Sirt1 inhibitor, EX-527 on neuronal viability 24 h after OGD for 2 h. n = 3. **d** Western blot evaluation of the protein levels of iNOS, SOD2/MnSOD, TNF-α, and pre-IL-1β and mature- IL-1β 24h after OGD. **e–h** Quantification of western blot data of iNOS, SOD2/MnSOD, TNF-α, and mature- IL-1β.∗∗*P* < 0.01 and ∗∗∗*P* < 0.001 vs. the control group. #*P* < 0.05 and ###*P* < 0.001 vs. the OGD group. n = 3

To ascertain the in vivo mechanism underlying the above-mentioned phenomenon, we constructed an in vitro primary neuron OGD model. Oxygen-sugar deprivation of primary neurons was achieved on the 7th day of culture in vitro for 2 h, followed by administration of different doses of CGA (0,100,200,300 and 400μM) after OGD. After 24 h of reoxygenation, neuronal proliferation activity was detected by the CCK8 assay. Results revealed that different drug concentrations did not affect neuronal cells under normal circumstances. Notably, after OGD, the proliferative activity of the cells was reduced to approximately 47.5%. By increasing the CGA concentration to 200,300μM, the cell proliferation activity was improved significantly. No significant difference in proliferative activity was reported at concentrations of 200 μM and 300 μM; as such, we selected 200 μM as the therapeutic concentration for in vitro experiments (Fig. 8a, b).

Through western blot, we detected the levels levels of pro-inflammatory factors IL-1β, iNOS, TNF-α, and antioxidant-related factors SOD2/MnSOD in the total protein of primary neurons. The experimental results revealed that, after oxygen-sugar deprivation, the levels of pro-inflammatory factors pre-IL-1β, mature-IL-1β, iNOS, and TNF-α increased significantly, while the levels of SOD2/MnSOD decreased significantly. Compared to the OGD group, the levels of pro-inflammatory factors pre-IL-1β, mature-IL-1β, iNOS, and TNF-α reduced significantly in the CGA (200μM) pretreatment group, while the levels of SOD2/MnSOD increased significantly. These data provide evidence on the neuroprotective effect of CGA by reducing inflammation and oxidative stress (Fig. 8d–h).

**Fig. 9:**
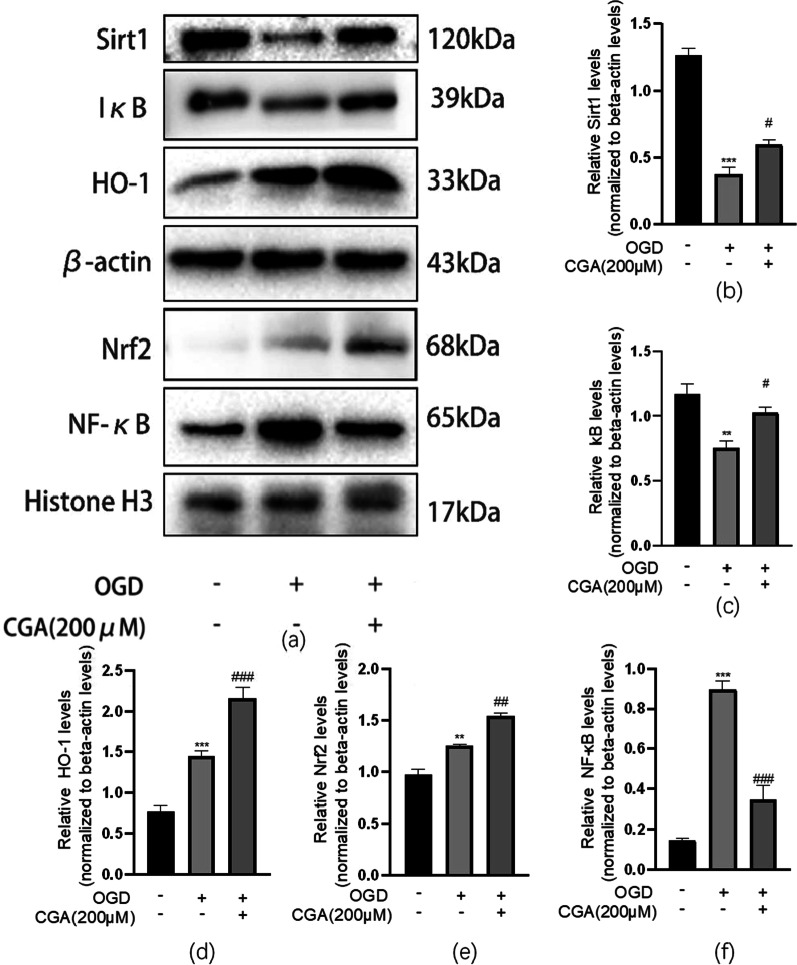
Chlorogenic acid regulates the Nrf2-NF-κB signaling pathway by activating Sirt1 in primary cortical neurons. **a** Western blot evaluation of the protein levels of Sirt1, IκB, HO-1, Nrf2, NF-κB 24h after OGD. **b–f** Quantification of western blot data of Sirt1, IκB, HO-1, Nrf2, NF-κB. ∗∗*P* < 0.01 and ∗∗∗*P* < 0.001 vs. the control group. #*P* < 0.05, ##*P* < 0.01 and ###*P* < 0.001 vs. the OGD group. n = 3

**Fig. 10:**
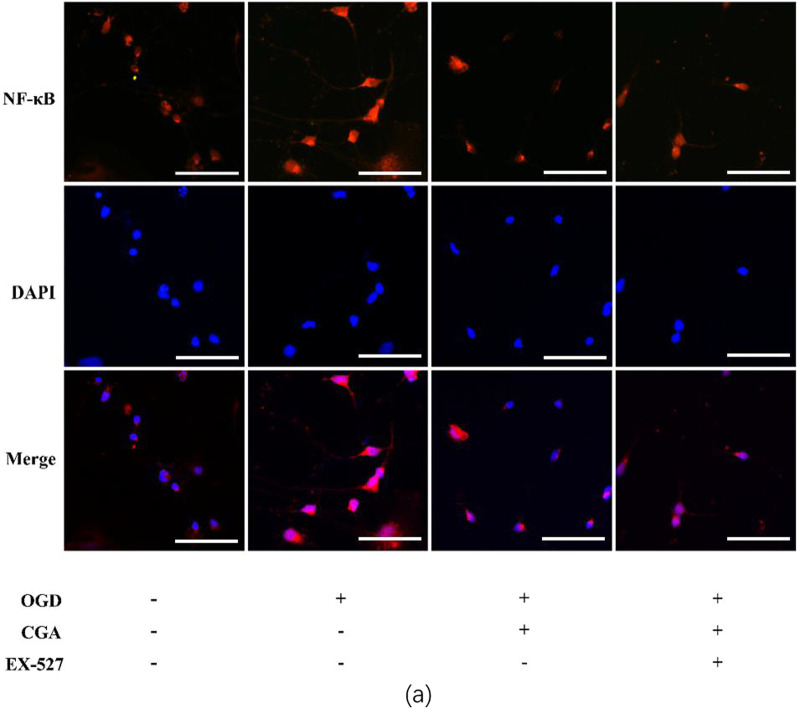
Chlorogenic acid regulates the Nrf2-NF-κB signaling pathway by activating Sirt1 in primary cortical neurons. **a** Representative immunofluorescent staining images for NF-κB expression in the primary neurons of the cortex. Scale bar = 25 μm. n = 3

Moreover, after CGA pretreatment, the levels level of Nrf2 in the nucleus was higher than that of the control group. And the levels level of NF-κB in the nucleus was significantly reduced in the CGA pretreatment group. The cell immunofluorescence results of NF-κB were consistent with the western blot experiment results of NF-κB in the nuclear protein in in vivo experiments (Fig. 9). The cytoplasmic protein test results showed that, after OGD, HO-1 was up-regulated, while the levels levels of Sirt1 and IκB were significantly reduced. However, CGA pretreatment significantly increased the levels level of HO1, Sirt1 and IκB (Fig. 10a–d). By introducing EX-527, a selective inhibitor of Sirt1, western blot detection of the levels of neuronal apoptosis-related proteins demonstrated a reversed anti-apoptotic effect of CGA through Nrf2-NF-κB (Fig. 10a, e–f). The cell viability analysis after pretreatment of primary neurons with EX-527verified that EX-527 reversed the protective effect of CGA (Fig. 8c).

## Discussion

Neonatal hypoxic-ischemic encephalopathy (HIE) is a type of perinatal asphyxia-induced brain damage. Although research on the pathogenesis of HIE has matured, mild hypothermia remains the sole clinical treatment for HIE. The short time window of mild hypothermia treatment and the limited nerve repair effect of severe HIE warrants an urgent exploration of an auxiliary treatment method for mild hypothermia to reduce HIE-induced nerve damage.

The research on the pathological damage mechanism of HIE covers cell inflammation, oxidative stress, apoptosis, autophagy, among others. It is of particular note that inflammation and oxidative stress are key players in HIE treatment. Studies by Serafina Perrone et al. demonstrated that hypoxic-ischemic injury activates various inflammation-related pathways, and a variety of inflammation-related cellular molecules, including cytokines (IL-1β, IL-6, IL-8, IL-10, TNF -α, etc) are activated after HI injury [39, 40]. Stress damage such as hypoxia and ischemia have been revealed to potentially cause significant changes in the levels of HO-1, HIF-1α, and other hypoxia-related proteins in brain tissue which were consistent with the results of the pro-inflammatory factors TNF -α and IL-1β in this experiment in the HI group. [39]. in vitro studies have revealed that the addition of the pro-inflammatory factor IL-1β inhibits the levels of HO-1 and can promote the occurrence of inflammation. Schipper et al. showed that up-regulation of HO-1 levels in the pathogenesis of multiple sclerosis (MS) was beneficial in maintaining the stability of the microenvironment in the cells and play a protective role after autoimmune neuroinflammation [41]. Nuclear factor erythroid 2 related factor 2 (Nrf2) is the upstream molecule of HO-1 and is activated by nuclear transfer [42]. Nerve injury causes up-regulation of Nrf2. Therefore, by regulating Nrf2 levels, the downstream cascade is activated to relieve the body's oxidative stress and inflammation [43]. In recent years, some anti-oxidative stress and anti-inflammatory drugs such as licochalcone A, Lycium ruthenicum polysaccharide 3 (LRP3) have been revealed to up-regulate Nrf2 intranuclear metastasis to exert neuroprotective effects, which is consistent with our present findings [44, 45]. NF-kB is an important nuclear transcription factor in cells. Kauppinen et al. found that inhibition of silent information regulator 1 (Sirt1) altered energy metabolism in cells, further stimulating the NF-κB-induced inflammatory response [46]. Our research results and the above research results can be mutually verified. At the same time, we compared the expression levels of multi-dimensional NF-κB and p-NF-κB in total protein, nuclear protein and cytoplasmic protein, and confirmed that chlorogenic acid could play a role by reducing the phosphorylation level of NF-κB into the nucleus.

Chlorogenic acid (CGA) is a phenolic antioxidant mainly extracted from honeysuckle. Previous evidence indicates that CGA exerts a neuroprotective effect in neurological diseases, including cerebral ischemia/reperfusion injury. For instance, Oboh et al. demonstrated that CGA exerted a neuroprotective effect in Alzheimer's disease by inhibiting the activities of acetylcholinesterase (AChE) and butyrylcholinesterase (BChE) and reducing the decomposition of acetylcholine and butyrylcholine [47]. Elsewhere, Liu et al. demonstrated that CGA regulated the Nrf2 pathway and further up-regulated Nrf2, NQO-1, and HO-1 to reverse cerebral ischemia/reperfusion-induced brain damage [27]. Shah et al. also found that CGA inhibited oxidative stress by reducing the level of reactive oxygen species (ROS), and further reduced focal cerebral ischemia-induced neuronal cell apoptosis [48]. Here, we, for the first time, proved that CGA exerts a neuroprotective effect on neonatal hypoxic-ischemic brain damage. Through in vivo and in vitro experiments, we have confirmed that CGA exerts anti-inflammatory effects by regulating Sirt1 to activate Nrf2-NF-κB, and further decreased the apoptosis of brain neurons. At the same time, we investigated the role of Sirt1 in the Nrf2-NF-κB pathway through intracerebroventricular injection in vivo and the addition of Sirt1 selective inhibitor EX-527 in vitro. Our analysis revealed that EX-527 reversed the aforementioned protective effects. Although our experiments did not prove the direct effect of chlorogenic acid and Sirt1, previous research reports have shown that there is a synergistic effect between polyphenols such as chlorogenic acid and Leu or HMB to activate Sirt1 [49]. In A549 cell apoptosis induced by paraquat, chlorogenic acid reduced apoptosis through Sirt1-mediated redox and mitochondrial function regulation [50]. Moreover, studies have shown that chlorogenic acid could resist oxLDL-induced endothelial cell apoptosis by up-regulating Sirt1 [51]. Chlorogenic acid can protect liver cells from palmitic acid-induced lipotoxicity by activating Sirt1 [52]. Through investigation of learning and cognitive functions, the results strongly demonstrated that CGA improves the spatial memory and learning and cognitive abilities of the HI-injured brain. The activation of astrocytes represents the proliferation of brain glial, which is a key contributor to brain tissue damage. Mounting evidence has also demonstrated the potential role of GFAP in the activation process of astrocytes in hypoxia, ischemia, and lipid peroxidation damage [53, 54]. Collectively, CGA plays a neuroprotective function in neonatal hypoxic-ischemic brain injury.

## Conclusion

In conclusion, our experimental research demonstrated that chlorogenic acid pretreatment exerted neuroprotective effects by significantly reducing cerebral infarction, decreasing the level of neuroinflammation and oxidative stress caused by HIBD, and moreover significantly reducing the activation of astrocytes caused by HIBD. Moreover, CGA could significantly improve the impairment of learning and cognitive function after HIBD in terms of long-term therapeutic effect. In terms of mechanism, the neuroprotective effect of CGA was mediated through the activation of Sirt1 regulating the Nrf2-NF-κB pathway. This study confirmed that CGA could be a potential treatment strategy for the treatment of HI brain injury.

## Data Availability

Not applicable.

## Abbreviations

HIE: hypoxic-ischemic brain injury
CGA: Chlorogenic acid
TTC: 2,3,5-triphenyltetrazolium chloride monohydrate
MWM: Morris Water Maze test
MDA: Malondialdehyde
CAT: Catalase
OGD: oxygen-glucose deprivation
CBP: cAMP-response-element-binding protein-binding protein
DMSO: Dimethylsulfoxide
CCK8: Cell-Counting Kit-8
DAPI: 4′,6-diamidino-2-phenylindole
BSA: Bovine serum albumin
